# Clinical Presentations and Surgical Features of Morgagni Hernia in Adults: A Retrospective Study

**DOI:** 10.1002/hsr2.70248

**Published:** 2024-12-09

**Authors:** Parviz Mardani, Farzad Dalfardi, Saman Bahmani, Rahem Rahmati, Fatemeh Zarimeidani, Bizhan Ziaian, Armin Amirian, Masoud Vafabin, Reza Shahriarirad

**Affiliations:** ^1^ Thoracic and Vascular Surgery Research Center Shiraz University of Medical Science Shiraz Iran; ^2^ Students Research Committee Shahrekord University of Medical Sciences Shahrekord Iran; ^3^ Department of Surgery Shiraz University of Medical Sciences Shiraz Iran; ^4^ School of Medicine Shiraz University of Medical Sciences Shiraz Iran

**Keywords:** adult hernia, congenital diaphragmatic hernia, Morgagni hernia, surgical repair

## Abstract

**Background and Aims:**

Morgagni hernias are rare congenital diaphragmatic defects that can lead to bowel obstruction and incarceration if not repaired. While this disease typically manifests as respiratory distress, frequent respiratory infections, and infant growth failure, it can sometimes be accidentally discovered in adulthood through cross‐sectional imaging. We aimed to report our experience managing this entity during 20 years at our referral center.

**Methods:**

A retrospective chart review was performed of adult patients diagnosed with Morgagni diaphragmatic hernia who underwent surgery at Shiraz University of Medical Sciences in two main referral hospitals between 2002 and 2022. Patients' demographic and clinical data, including surgical features and hospitalization, course were extracted and subsequently analyzed.

**Results:**

Seventeen patients, including three men and 14 women, with an age range of 26 to 92 years (average age 61.5 years), were diagnosed with Morgagni diaphragmatic hernia. Three patients had left‐sided hernias, and 14 had right‐sided hernias. The most common clinical manifestations were shortness of breath and abdominal pain. The most commonly herniated contents were the omentum and colon. All patients underwent laparotomy, and the hernia sac was removed in 11 patients.

**Conclusions:**

Morgagni is a rare congenital diaphragmatic hernia usually diagnosed incidentally. Laparoscopic repair has high success rates and is a viable option for patients with this pathology.

## 1 Introduction

In 1769, an Italian anatomist and pathologist named Giovanni Morgagni reported a congenital defect called a Morgagni hernia (MH) [1]. It manifests as a hernia through an anterior retrosternal diaphragmatic defect between the sternum's xiphoid process and the muscle's costal portion [2]. The cause and pathophysiology of MHs seem to be uncertain [3]. Although the Morgagni opening is usually congenital, it can also be acquired [4, 5]. There's also the possibility that the hernia develops early on, but remain asymptomatic [6]. In adults, MH accounts for only 2–5% of cases, and the exact acquired incidence is unclear [7, 8]. The congenital incidence is predicted to be one in 2000–5000 [5, 9].

MHs are more often found on the right side, mostly featuring a hernia sac [5, 7, 10]. The contents of the hernia sac and size determine if a patient is symptomatic or asymptomatic [7, 8, 9, 11]. MH is often found accidentally or intraoperatively during regular imaging to evaluate other conditions [3, 8]. A timely diagnosis may be prolonged since patients frequently report nonspecific symptoms of respiratory and gastrointestinal problems [12, 13, 14].

The presentation, physical examination, chest or abdominal radiography, and eventually, computed tomography (CT) scan without contrast all contributed to the patient's suspected diagnosis of MH [4, 8, 15]. A chest radiograph is usually sufficient for a radiological diagnosis, whereas a chest computed tomography (CT) scan may reveal the defect with up to 100% accuracy [5]. Surgical treatment due to the risk of strangulation is indicated for all patients of MH, even those with no symptoms [3, 4, 16]. Repair methods and strategies are still a matter of some controversy [5]. Both abdominal and thoracic open and minimally invasive techniques are capable of treating hernias [2, 3, 17]. Surgeon judgment plays a key role since there is no agreement on a conventional surgical procedure due to the rarity of the entity [2, 4].

Although long‐term outcomes of surgical repair and the risk of recurrence are still unclear [2, 5], the patients typically completely recover following repair with no traces of their preoperative symptoms [5]. Since MHs are rare in adulthood, this study aimed to assess the prevalence, symptoms, hernial condition, surgical management, and complications to enhance the understanding of MH.

## 2 Methods and Materials

A retrospective case series study was conducted on adult patients diagnosed with Morgagni diaphragmatic hernia who underwent surgery at two main referral hospitals (Namazi and Bu Ali) affiliated with Shiraz University of Medical Sciences for 20 years (2002–2022).

All patients underwent plain chest X‐rays and relative procedures based on their clinical features, including barium swallow test, and patients suspected of other abdominal pathologies underwent an abdominal ultrasound. Patients with gastrointestinal symptoms underwent endoscopy. The diagnosis was based on clinical features and confirmation radiographic imaging with a CT scan.

The patients were treated with a high midline incision laparotomy, and during exploration of the abdominal cavity, the context herniated into the sac is reduced, and the sac is resected with a primary repair using a Nylon 1.0 loop suture in the diaphragm.

Patient information, including age, gender, initial presentation, laterality, hernia sac presence, clinical tests, radiographs, imaging reports, and surgical technique, were documented and subsequently analyzed. Pediatric patients (age under 18) and incomplete hospital records were excluded. The patients were followed during their routine visits at the clinic for any occurrence of morbidity, mortality, or recurrence for 1 year. Data were entered into SPSS version 26.0 (IBM, USA). Descriptive data are reported as mean, standard deviation (SD), frequency, and percentage (*n*, %).

## 3 Results

### 3.1 Demographic and Population Features

During the 20 years of our study, 302 patients with congenital diaphragmatic hernia and 410 patients with diaphragmatic hernia were diagnosed. Among these patients, 43 were operated on with a diagnosis of Morgagni diaphragmatic hernia; however, 26 were excluded due to age under 18, leaving us with a total remaining number of 17 cases who were evaluated in our study.

The 17 patients included in our study are reported in Table 1. As demonstrated, our patients' age ranged from 26 to 92 (mean: 61.5; SD: 20) and mainly consisted of females (*n* = 14; 82.4%). The majority of MHs were on the right side (82.4%).

**TABLE 1:** The demographic and clinical features of adult patients diagnosed with Morgagni diaphragmatic hernia operated at referral hospitals in Shiraz, Iran, during 2002–2022.

Patient's No.	Age/Sex	Site	Symptoms	Comorbidities	Content of hernial sac	Width of hernial sac [cm]	Operative treatment	Hospital stay
1	87/Female	Right	Epigastric pain + GiB (coffee ground vomit)	Hypertension	Stomach + Transverse colon	—	Laparotomy + Repair of MH	17
2	76/Female	Right	Dyspnea	Chronic kidney disease; heart failure (EF = 30%)	Omentum + Transverse colon	4 × 4	Laparotomy + Repair of MH	11
3	64/Female	Right	Abdominal pain + Constipation	Hypertension	Omentum + Transverse colon	6 × 6	Laparotomy + Repair of Rt side diaphragm	4
4	40/Female	Right	Abdominal pain + Obstipation + Dyspnea	Scoliosis, Peptic uwith GIB, PsHx of thoracoscopic repair of hiatal hernia	Omentum + Large bowl + Small bowl	6 × 8	Laparotomy + Reduction of volvulus + Pneumolysis + Adhesionolysis + Repair of three sites of bowel deserosation + Repair of MH + Mesh insertion + Right side chest tube insertion	11
5	41/Male	Right	Dyspnea + Cough	DM. COPD (Smoker, Opium addict), HCV + Peptic dis. (GERD)	Omentum + Large bowl + Small bowl	—	Laparotomy + Repair of MH + Mesh insertion	12
6	54/Female	Right	Dyspnea	Smoker, mild Disk herniation	Omentum + Large bowl + Small bowl	—	Laparotomy + Repair of MH + Omentectomy + Hemovac insertion + Chest tube insertion	6
7	70/Female	Right	Sudden unset Dyspnea	COPD (smoker), HTN	Omentum + Large bowl	—	Laparotomy + Repair of MH + Partial omentectomy + Hemovac drain insertion	15
8	77/Female	Right	Chronic postprandial Dyspnea + Chest pain	AF, RA, HTN	Omentum	3 × 5	Laparotomy + Repair of MH + Hemovac insertion	10
9	75/Female	Right	Dyspnea + Epigastric pain	Constipation	Omentum	—	Laparotomy + repair of MH	3
10	72/Female	Right	Epigastric pain	Anemia	Incarcerated omentum	3 × 3	Laparotomy + repair of MH	5
11	59/Male	Left	Epigastric pain + previous abdominal trauma	—	Stomach	5 × 5	Laparotomy + wedge resection of mass of stomach + Repair of MH at the left side by mesh + Left side chest tube insertion	—
12	45/Female	Left	Abdominal pain + Dyspnea + Partial obstruction	—	Incarcerated omentum	—	Laparotomy + Omentectomy + Reduction of MH sac + Repair of MH defect + Entrolysis	5
13	26/Female	Right	Postprandial Dyspnea + Cough + Abdominal pain + Dysphagia	—	Right colon + Transverse colon	6 × 6	Laparotomy + Repair of MH	3
14	66/Female	Right	Dysphagia + Dyspnea + Chest pain	HTN	Omentum	6 × 6	Laparotomy + Repair of MH	7
15	76/Female	Right	Dysphagia + Dyspnea + Abdominal pain + N/V	HTN	Omentum	6 × 6	laparotomy + Repair of MH + Right hemi‐colectomy + Ileocolon end to side anastomosis	—
16	26/Male	Left	Incidental finding	HTN, IHD	Omentum	—	Laparoscopy reduction of herniated omentum to abdominal cavity + laparotomy and partial omentectomy + repair of MH	4
17	92/Female	Right	GiB (Coffee ground vomiting) + Abdominal pain	—	Omentum + Transvers colon	—	Laparotomy + Reduction of herniated omentum and colon to abdominal cavity + resection of the sac of hernia + repair of MH	4

Abbreviations: GiB, gastrointestinal bleeding; MH, Morgagni hernia; N/V, nausea and vomiting.

### 3.2 Presentation Features

Five (29%) of our patients were asymptomatic at the time of the visit, but in the past, they had nonspecific symptoms such as postprandial chest and abdomen discomfort. The most common clinical manifestations were shortness of breath (especially postprandial), abdominal discomfort and pain, dysphagia, nausea and vomiting, chest pain and discomfort, cough, bloody vomiting, constipation, and intestinal obstruction symptoms. Most of the patients chronically had nonspecific clinical symptoms intermittently; however, one of the patients (No 7) came to the hospital complaining of sudden shortness of breath. Also, one of the patients (No 5) who complained of shortness of breath and cough, had a history of intermittent episodes of gastrointestinal bleeding. One of the patients (No 16) developed shortness of breath sometime after contracting the flu, and a CT scan was performed to check for lung involvement, which was incidentally diagnosed as an MH.

**FIGURE 1:**
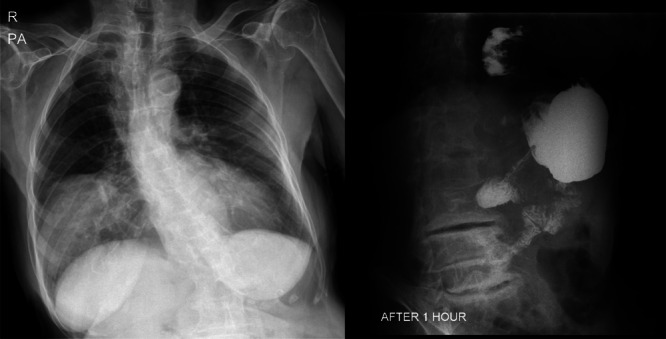
X‐ray and upper gastrointestinal series scan of an 87‐year‐old female patient. Large fat density is seen in the lower half of right hemithorax in favor of a large diaphragmatic hernia. Contrast study demonstrates the antrum and stomach and distal part of duodenum is displaced to the lower part of chest, in favor of the mentioned hernia.

Patients with gastrointestinal symptoms underwent endoscopies, including two patients who complained of repeated vomiting of blood, and underwent endoscopies (No 1 and 17). One of these patients (No 1) was hospitalized with a complaint of vomiting blood. After initial investigations, a diagnostic endoscopy was performed for the patient due to the presence of irregularity in the pyloric mucosa, with the suspicion of malignancy; a biopsy was performed for the patient, which was negative. The patient was put on protein pump inhibitor (PPI) treatment; however, during endoscopies, the prob was reported to have not passed the D1–D2 junction due to repeated gastrointestinal bleeding. Therefore, she underwent a CT scan to rule out malignancies, in which MH was detected (Figure 1).

Among our patients, only one (No 11) had a history of blunt trauma to the abdomen.

### 3.3 Radiographies and Imaging

Figure 1 demonstrates the X‐ray and upper gastrointestinal series scan of this patient (No 1), which revealed a large fat density in the lower half of the right hemithorax in favor of a large diaphragmatic hernia. Also, a contrast study demonstrated the antrum and stomach and distal part of duodenum is displaced to the lower part of chest, in favor of the mentioned hernia. Significant delay passage of contrast was also seen.

**FIGURE 2:**
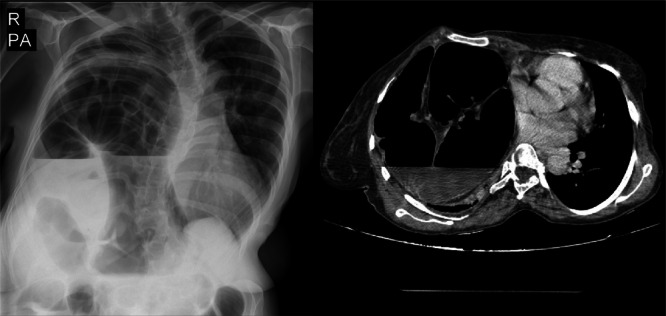
X‐ray and computer tomography scan of a 40‐year‐old female with Morgagni hernia.

Figure 2 demonstrates the CT and X‐ray of patient No 5 in our study, demonstrating a large diaphragmatic hernia due to a large defect at anteromedial aspect of the right hemidiaphragm. The mentioned defect is about 100 mm in length. Findings are in favor of a Morgagni hernia. There is herniation of bowel loops which are most likely part of the colon (hepatic flexure) into the defect. The mentioned bowel loops significantly dilated measuring up to 90 mm in diameter.

**FIGURE 3:**
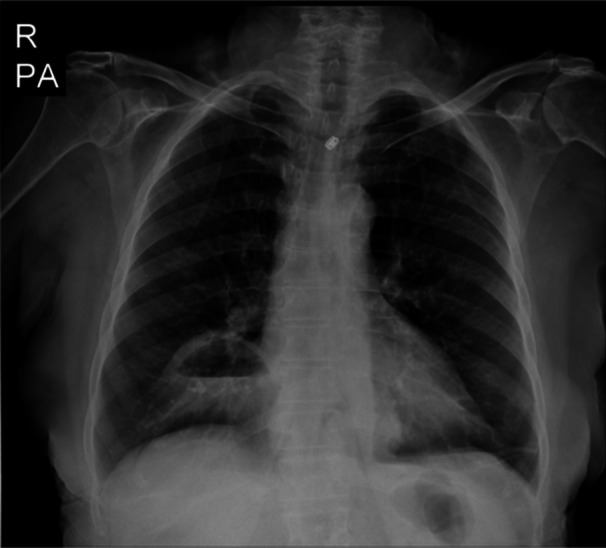
X‐ray of a 77‐year‐old female with Morgagni hernia.

**FIGURE 4:**
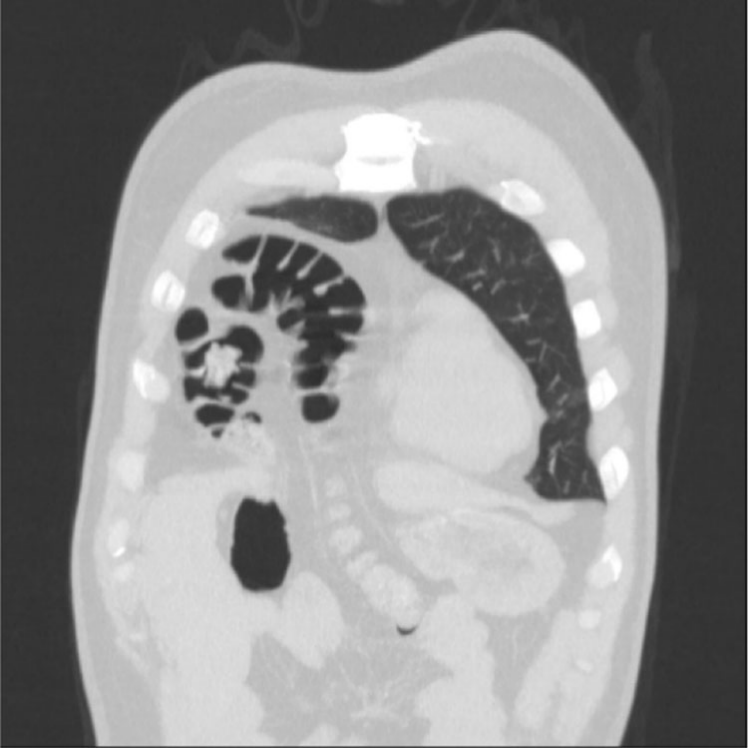
Computed tomography scan of a 41‐year‐old male patient with Morgagni hernia (Coronal cut).

Figure 3 demonstrates the X‐ray of patient No 9, and Figure 4 demonstrates the coronal CT scan of patient No 6. The CT scan demonstrated a large defect in the anterior aspect of the right hemidiaphragm measuring about 65 × 35 mm associated with herniated mesenteric fat and hepatic flexure of the colon without sign of fat stranding or obstruction.

### 3.4 Hernia Features

The most common herniated contents in these patients were the omentum, colon (especially transverse colon), small intestine, and stomach. Gastric volvulus was observed and corrected in one case (No 4). In two cases (No 10 and 12), the omentum was observed inside the hernia. The hernia size ranged from 3 to 8 (average 5.4; SD: 1.4) cm. In the remaining patients (Table 1), the size of the hernia was described as “large.”

### 3.5 Surgical Features

All patients underwent laparotomy; however, in one patient (No 16), laparoscopy was initially performed, and after removing the contents of the hernia, the surgical incision was changed to laparotomy due to insufficient visibility.

The repair method is based on the surgeon's preference and the degree of satisfaction with the quality of the repair. Among our cases, 15 patients (88%) underwent hernia repair with sutures (nylon loop 0 or 1). In one patient (No 11), a hernia was repaired with mesh over the diaphragm defect, and two patients underwent hernia repair with mesh‐reinforced sutures (No 4 and 5). Also, a hernia sac was removed in 11 (64.7%) patients. In two patients, the entire omentum was removed, and in three patients, a part of the omentum was removed. Also, the chest tube was implanted in two (11.8%) patients after hernia repair.

**FIGURE 5:**
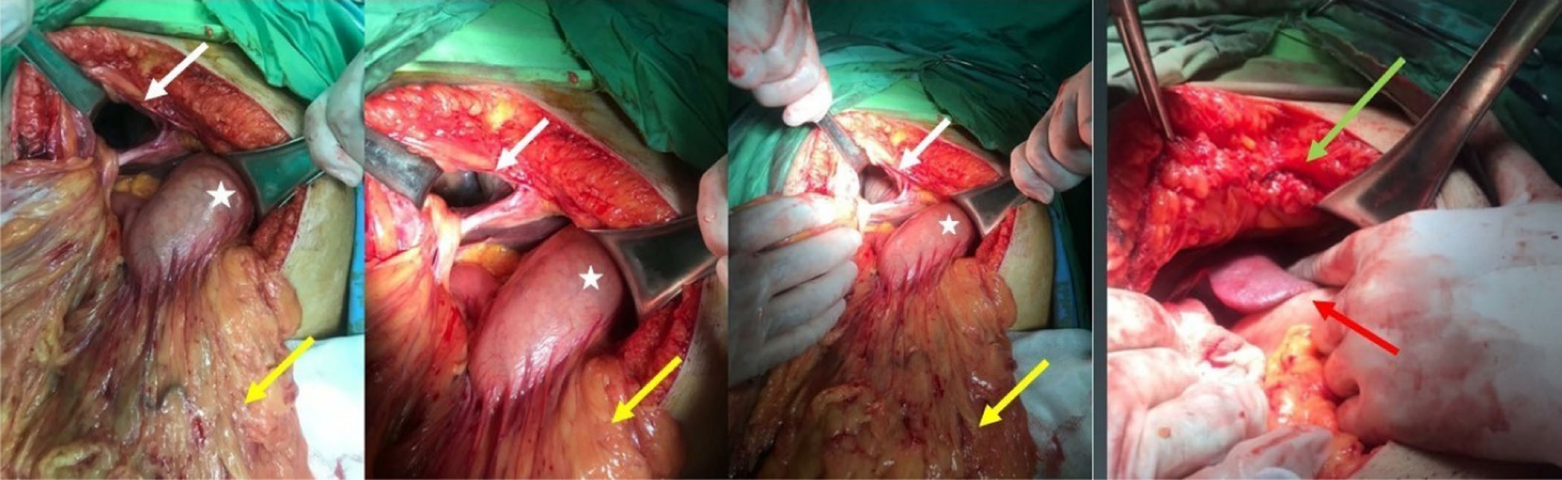
Intraoperative image of a 76‐year‐old female patient with a right Morgagni hernia undergoing laparotomy. The omentum (yellow arrows) was extracted from the hernia sac and the chest cavity (white arrows) and subsequently repaired (green arrow); star: stomach; red arrow: liver.

Figure 5 demonstrates the intraoperative image of one of the patients in our study (No 3).

### 3.6 Postoperative Features

The number of days the patients stayed in the hospital varied from 3 to 17 days (average 7.8 days). Also, no recurrence of hernia has been reported in any of the patients, and all patients were discharged from the hospital in good general condition.

## 4 Discussion

Over 20 years, we studied patients with MH to gain insight into their condition and assist surgeons in providing better, more adequate care. We reviewed 17 patients from two referral hospitals. Middle‐aged females were more diagnosed with MH presented by dyspnea or gastrointestinal problems. All of our patients were nonemergency and underwent laparotomy with 88% primary repair and 65% removal of the hernial sac. No 30‐day mortality and serious complication or recurrence were seen during the 6‐month follow‐up.

Consistent with earlier studies showing female preponderance and 47 to 67 median age range of the adult MH patients, 82.4% of our patients were female with an average age of 61.5 years [3, 4, 18, 19]. Also, case No 16 supports that hernia‐related problems are more common in males at an earlier age [3]. Additionally, individuals with elevated intra‐abdominal pressure settings, including pregnancy, trauma, obesity, chronic cough, and chronic constipation, are more prone to MH [3, 4, 5, 10]. Regarding this, 24% of our patients also reported experiencing chronic cough and/or constipation, and just case No 11 reported abdominal trauma history. Obesity, multiparity, chronic obstructive pulmonary disease, and constipation, were observed in 50% of MH patients in short‐series research by Tarcoveanu et al. as well [17]. Therefore, middle‐aged females with intra‐abdominal pressure conditions are more suspicious of MH.

Herniation into the left hemithorax is still conceivable, especially in cases with pericardial abnormalities in proximity to the Morgagni foramina, but the preference for right‐sided presentation is due in large part to the protective blockage of the left sternocostal triangle by the overlying pericardium [2, 3]. This study's findings of 82.4% right‐sided MH and 17.6% left‐sided are consistent with those of previous reviews and subsequent studies, which reported a prevalence of 75%–90% right‐sided MH and 11%–22% left‐sided MH [3, 4, 8, 18]. Nevertheless, no bilateral MH was detected throughout our 20‐year observation, although reviews report a prevalence of 3% to 5% [3, 4]. Except for longer hospital stay for left‐sided MH patients, surgical approach and defect size were insignificantly associated with hernia location [4].

Previous research has shown that up to 50% of patients can present with no symptoms [4, 8, 18]; however, we identified 29% as asymptomatic, and Agalar et al. reported no asymptomatic individuals [9]. This might be caused by the improved screening and the availability of diagnostic approaches. Furthermore, similar to our only incidental MH (No 16), available research showed that men were more likely to be asymptomatic [2]. According to the systematic review, preoperative diagnosis was made in about 78% of the asymptomatic group; while the intraoperative diagnosis was around 22.4% [4]. Similar to previous studies, we identified dyspnea, particularly postprandial, as the most frequent initial symptom among symptomatic patients [3, 18]. The percentage was 65%, greater than the previously reported 33–58% [3, 4, 5, 6, 11, 18, 19]. We also added evidence (No 7) to the previous report of sudden onset dyspnea following MH [6]. Following, abdominal pain was seen in 39–83% of patients [3, 4, 5, 11, 18, 19, 20]. In our study, 59% of our right and left‐sided MH patients manifested hernia with abdominal pain. Other earlier reported symptoms were reflux, nausea and vomiting, cardiac symptoms like chest pain or palpitation, dysphagia, bowel obstruction, early satiety, weight loss, and gastrointestinal bleeding [4, 5, 11, 18, 19]. This finding shows that early warning indications of herniation could present with respiratory and/or gastroesophageal problems, even suddenly.

A hernia can lead to a compromised blood supply to prolapsed organs, which changes in abdominal pressure could intensify. Vascular impairment due to organ strangulation could cause mild to severe pain [3]. Aligned with findings from systematic reviews and subsequent investigations, the omentum (82%) and colon (53%) were the most commonly detected contents within the hernia sac in our study [4, 18]. While 17.4% with a herniated liver were reported in earlier studies, we did not see any liver involvement [4]. Additionally, our 18% small intestinal herniation is higher (0–15.8%), and 12% stomach herniation is lower (16–20%) than earlier studies [4, 18]. We experienced just two cases of incarcerated omentum in our study (11.7%); while other studies have reported a rate of 7.7% of previous MH cases related to hernias were incarcerated, and 3.5% were attributed to strangulation [4]. Besides, although previous studies have reported that 27.2% of MH patients needed an emergency procedure, we did not have any such cases to report, similar to Young et al. study [2, 4]. Surgeons must be aware of the risks to all intra‐abdominal organs and carefully assess patients with abdominal pains. Patients with clinical suspicion of strangulation should have a full workup since missed herniation can be harmful.

Surgery is the fundamental basis for treating MH; however, data on the efficacy of various surgical approaches and outcomes are limited [11]. Both the abdominal (laparotomy and laparoscopy) and thoracic (thoracotomy and thoracoscopy) approaches are considered when performing hernia repair surgery [5]. According to an earlier systematic review, more than half of the patients had open surgery. Furthermore, the initial minimally invasive procedure had to be changed to open surgery for one out of every 100 patients, similar to the No 16 case [4]. Open surgery promotes hernia reduction, allows for better functional capacity and flow assessment, and is more capable of finding a bilateral Morgagni defect [9, 11, 21]. In this regard, the abdominal approach is linked to shorter hospital stays [4]. Alongside the median duration of stay for open surgery was 6.8 days [4, 22], our patients were hospitalized for 7.8 days on average.

In this study, a minimally invasive approach was used for one case, only as a diagnostic procedure. While minimally invasive surgery is becoming a growing trend, earlier studies predictably, show a higher number of cases treated with an open technique. For adults with a MH, the results of systematic reviews suggest that an open approach is better for emergent surgery, while laparoscopic surgery is associated with a shorter hospital stay [4, 23]. However, when it comes to perioperative morbidity and mortality, both approaches showed comparable findings [4]. Nevertheless, there is still no standardized technique.

In our center, the main surgical method was open surgery due to the emergent nature of the cases, the surgeon preference, and also the limited equipment and resources. There are several reasons why low‐income and developing countries have a lower rate of minimally invasive surgery. One obstacle to the widespread use of minimally invasive surgery is the underinvestment in surgical equipment and training and simulation labs, caused by limited financial resources. It is further exacerbated by the high volume of patients and operations in referral centers. The lack of thorough health and social security policies, along with the ineffective use of available resources, makes these challenges worse [24, 25, 26]. There might be an upgrade toward more minimally invasive surgeries with fewer complications and shorter hospital stays if human, financial, and educational resources were well‐managed.

Moreover, primary closure, mesh interposition, and mesh strengthening with primary closure are all surgical methods used to repair hernias [21]. According to Katsaros et al. systematic review, the mesh is most often used in minimally invasive operations (76.9%), whereas most suturing is done in open surgery (69.9%) [4]. Most surgeons chose mesh in retrospective reviews of Morgagni defects bigger than 5 cm [1, 6, 11]. Our patients' mean diaphragmatic defects were less than the 7.5–8.8 cm reported in two prior reviews [3, 4], but they were still slightly larger than 5 cm. Hence, our surgeons preferred primary closure in 88% of patients with no consequent complications.

Moreover, the MH almost always consists of a hernial sac [9]. Although our surgeons prefer to remove the whole sac in 65% of cases, only 16.1% of hernia sacs removal reported in a recent systematic review [4]. There is an ongoing discussion on whether or not it is necessary to remove the hernia sac during the repair process. While some argue that leaving it intact does not increase the risk of complications or recurrence, others recommend its total excision to enhance diaphragm reattachment. Nevertheless, studies have found that keeping the sac after surgery can lead to postoperative symptoms [3, 9, 10, 19]. There is also a debate surrounding the removal of the sac, as it may harm crucial intraoperative organs like the lungs, pericardium, and mediastinum. Although 20% or less of patients had postoperative problems after sac excision, which were either significant or mild, it seems that sac removal by the expert surgeon is the better idea [4, 18].

Earlier studies have shown a recurrence rate as high as 42%, whereas others and we have found no recurrences with either minimally invasive or open surgery [5, 18, 19]. Although it is unknown what factors contribute to the varying rates of recurrence across different types of repairs, closing the defect under tension without a patch, not detaching the sac, repairing with absorbable suture, and having a past medical history of Down syndrome are all risk factors for recurrence [5, 21]. Besides, numerous studies indicated no statistical significance of perioperative morbidity and mortality between different surgical repairs [2, 4, 22]. However, common complications were incisional or port site hernias, cardiac tamponade, pulmonary problems, and wound seroma or hematoma [2, 4, 6, 9, 16, 22, 27]. Additionally, although a previous review reported a 30‐day mortality of 2.3% for MH, no 30‐day mortality was seen in our study [4]. Concerning recurrence and complications, routine follow‐up to monitor the patient, particularly high risks, is critical after the operation.

## 5 Conclusion

Right‐sided diaphragmatic MH is more frequent in middle aged females and is an uncommon type of congenital diaphragmatic hernia. Patients are asymptomatic or often have pulmonary and gastrointestinal problems. Strangulation is a medical emergency necessitating surgical interventions. There is no broadly agreed method for the type of surgery and removing a hernia sac; hence the surgeon's choice depending on the individual patient is usually respected more. Therefore, further comprehensive and larger research on MH management is required.

## 6 Strength and Limitation

Our research has some limitations. Most included studies had very small patient samples and potential reporting bias. In addition, long‐term postoperative outcomes like hernia recurrence are generally underreported due to insufficient long‐term follow‐up. When evaluating the efficacy of various treatment plans and surgical procedures, it is essential to account for these factors. Finally, since our data came from just two emergency departments, it is possible that our findings do not generalize to patients in other settings. To confirm these findings, prospective, multicenter longitudinal research is necessary.

## Author Contributions

**Parviz Mardani:** conceptualization, methodology, supervision, data curation. **Farzad Dalfardi:** data curation, supervision. **Saman Bahmani:** data curation. **Rahem Rahmati:** writing–original draft. **Fatemeh Zarimeidani:** writing–original draft. **Bizhan Ziaian:** methodology, supervision. **Armin Amirian:** writing–review and editing, methodology. **Masoud Vafabin:** writing–review and editing. **Reza Shahriarirad:** data curation, project administration, investigation, writing–original draft, writing–review and editing.

## Ethics Statement

The data acquisition and analysis of the present study were approved by the Medical Ethics Committee of Shiraz University of Medical Sciences (IR. SUMS. MED. REC.1401.405) and are in accordance with the amended Declaration of Helsinki. All experiments were performed in accordance with relevant guidelines and regulations. Based on the retrospective nature of the study, the informed consent waiver was approved by the Ethics Committee of Shiraz University of Medical Sciences. Patients' information was anonymized before analysis, and confidentiality was assured by the researcher. The lead author, Reza Shahriarirad, affirms that this manuscript is an honest, accurate, and transparent account of the study being reported; that no important aspects of the study have been omitted; and that any discrepancies from the study as planned (and, if relevant, registered) have been explained.

## Conflicts of Interest

The authors declare they have no conflicts of interest.

## Transparency Statement

The lead author Reza Shahriarirad affirms that this manuscript is an honest, accurate, and transparent account of the study being reported; that no important aspects of the study have been omitted; and that any discrepancies from the study as planned (and, if relevant, registered) have been explained.

## Data Availability

All data generated or analyzed during this study are included in this published article.
